# Digital Doses: Virtual Reality Use for Perioperative Pain and Anxiety in Patients Undergoing Hand Surgery

**DOI:** 10.1016/j.jhsg.2025.100830

**Published:** 2025-09-23

**Authors:** Angel X. Xiao, Brian Chen, Christopher Chiong, Nicholas H. Lee, Igor Immerman, Sakura Kinjo

**Affiliations:** ∗ UCSF Department of Orthopaedic Surgery, 521 Parnassus Ave, San Francisco, CA; † Rosalind Franklin University, 3333 N Green Bay Rd, North Chicago, IL; ‡ Touro University California, 1310 Club Dr, Vallejo, CA; § UCSF Department of Anesthesia and Perioperative Care, 521 Parnassus Ave, San Francisco, CA

**Keywords:** Outpatient hand surgery, Perioperative, Virtual reality

## Abstract

**Purpose:**

Virtual reality (VR) is increasingly recognized as a complementary tool to address pain and anxiety. We conducted a randomized controlled trial to evaluate the effectiveness of VR for the management of pain and anxiety in patients undergoing minor hand surgery.

**Methods:**

Patients undergoing outpatient hand surgery were randomized to VR or control groups. In addition to the standard anesthetic protocol, the VR group received a VR experience as part of their preoperative care. Patient anxiety and pain scores were collected using the Numerical Visual Analog Anxiety Scale and Numerical Rating Scale, respectively. In addition, we recorded changes in patient hemodynamics and any additional medication doses required to manage pain or anxiety.

**Results:**

Forty-one patients (21 VR and 20 control) were enrolled. There were no differences in reported pain or anxiety scores before, during, or after surgery. There was no difference in vital signs or recovery times. Patients in the VR groups received less additional midazolam (0.4 mg vs 1.2 mg) and fentanyl (10 mcg vs 27.4 mcg) compared with patients in the control group. In a multivariable model, VR use remained the only significant predictor for no required midazolam. Eighty-five percent of patients believed that the use of VR positively impacted their surgical experience. As a result of the VR experience, 78% believed that their anxiety decreased and 61.1% believed that their pain decreased.

**Conclusions:**

Although patient pain and anxiety levels between the VR and non-VR groups were similar, the VR group required significantly less midazolam and fentanyl. Moreover, VR use was the only predictor of not requiring midazolam administration during surgery. Patient satisfaction was high with VR usage. VR implementation during minor hand surgery is a viable option to improve patient experience.

**Type of study/Level of evidence:**

Therapeutic IIB.

Preoperative anxiety is a commonly reported phenomenon, affecting between 59% and 92% of patients undergoing elective surgery.^1^, ^2^, ^3^ Anxiety and fear related to anesthesia and surgery have been linked to a physiologic response including tachycardia, hypertension, nausea, as well as increased intraoperative and postoperative pain.^4^^,^^5^ Within the perioperative setting, anxious patients often require more anesthesia and additional doses of analgesics.^6^^,^^7^ These medications are associated with potential adverse effects such as drowsiness, respiratory depression, and nausea, which can both negatively affect the patient’s experience as well as lead to prolonged recovery times. Moreover, within the last two decades, opioid abuse and associated deaths have increased. As a result, there is growing interest in identifying safer and more cost-effective alternatives for managing surgical anxiety and pain.^7^, ^8^, ^9^ Specifically, there has been increased research on nonpharmacological methods to target anxiety and pain, such as relaxation techniques and visual distraction.^9^^,^^10^

Virtual reality (VR) has emerged as a powerful technology that has been effectively used in health care settings to treat anxiety and pain with minimal noted side effects.^11^^,^^12^ In an immersive VR environment, a head-mounted display projects simulated images to the user that allow for user immersion and interaction with a virtual world. VR technology represents an advanced innovation that offers considerable applications beyond entertainment including psychiatry, rehabilitation, and education.^13^, ^14^, ^15^ VR use in medical settings as a complementary tool to traditional pharmacological approaches has gained traction in outpatient procedural fields such as dentistry, wound care, and bedside pediatric procedures, in which it has been shown to enhance patient comfort and reduce both procedural anxiety and pain.^16^, ^17^, ^18^ Exposure to distraction to stimulate the visual cortex and promote cognitive engagement has been hypothesized as a possible mechanism for VR’s ability to limit the user’s processing of pain and stress signals.^19^

Despite the emerging adoption of VR within health care, there is a lack of research surrounding the use of VR to target patient pain and anxiety in the perioperative setting. The aim of this study is to evaluate the effectiveness of VR in reducing perioperative anxiety and pain among patients undergoing minor orthopedic hand surgery. We plan to measure pain and anxiety directly using validated scales and patient self-reported satisfaction surveys as well as indirectly via hemodynamic measures and perioperative medication use. We hypothesize that a reduction in preoperative anxiety may result in decreased patient need for anxiolytics or analgesics and, ultimately, lead to increased patient satisfaction.

## Methods

**Figure 1:**
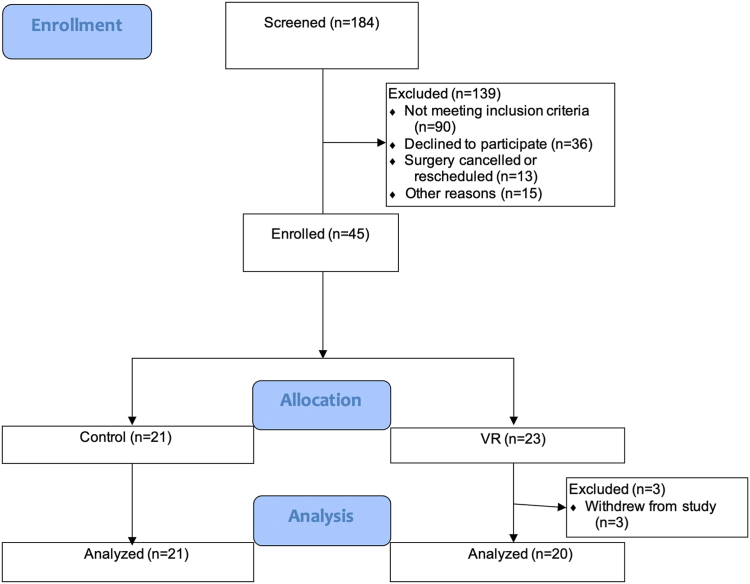
Consort diagram.

This was a prospective randomized controlled trial (RCT) conducted between January 2019 and July 2023. The study was registered at www.clinicaltrials.gov (NCT03744845 The Use of Virtual Reality to Reduce Anxiety and Pain in Perioperative Settings). The study adhered to the Consolidated Standards of Reporting Trials reporting guidelines, and the protocol is shown in Figure 1. This study was approved by the Institutional Review Board (IRB # 17-22021).

The study was conducted in a high-volume orthopedic ambulatory surgery center (ASC) affiliated with an academic hospital. All patients undergoing minor hand procedures, defined as procedures projected to be < 2 hours long, by fellowship-trained hand surgeons (I.I. and N.H.L.), were enrolled. Participants were adults aged 18–75 years, classified as American Society of Anesthesiologists (ASA) physical status I–II and English-speaking. Exclusion criteria included ASA physical status >III, allergies to fentanyl, midazolam, or propofol, history of seizures or migraines, history of chronic pain syndrome, history of high-dose or long-acting opioid use (daily oral morphine equivalents of ≥60 mg), or history of severe anxiety managed with daily anxiolytics. Patients with psychiatric comorbidities or physical disabilities that preclude VR use were also excluded.

Participants were evaluated at the anesthesia preoperative clinic prior to surgery to determine eligibility. Eligible patients were contacted by phone or on the day of surgery. Informed consent was obtained in the preoperative area on the day of surgery. All patients in this study received a combination of local anesthetic and intraoperative monitored anesthesia care (MAC). Buffered 1% lidocaine with epinephrine was injected in all patients in the preoperative holding area prior to administration of the VR device. All cases performed under a tourniquet used a forearm tourniquet.

**Figure 2:**
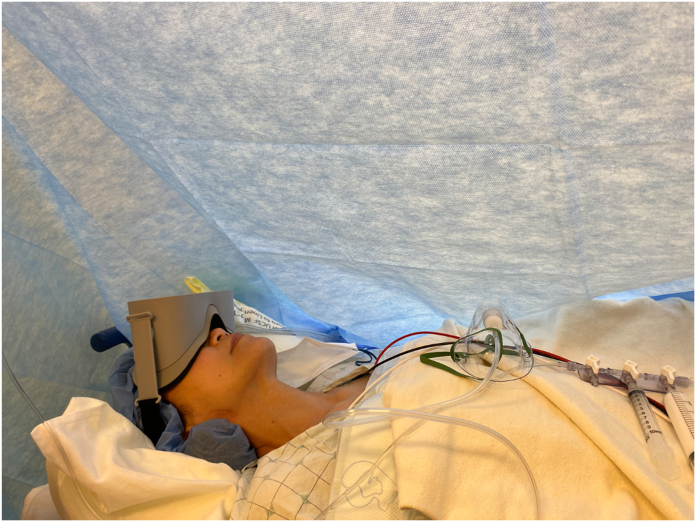
Intraoperative setup for a patient in the VR treatment group.

The anesthesia plan was created by one of our ASC anesthesiologists (S.K.) and was detailed to all participating anesthesia providers (Figure S1, available online on the *Journal’s* website at https://www.jhsgo.org). The control group received standard perioperative care, including administration of midazolam, fentanyl, and propofol as needed. Patients were instructed that they can request medications as needed, and that no medications would be administered unless requested. The anesthesia provider was instructed to ask the patients how they were doing every 15 minutes but was not allowed to directly offer medications to avoid suggestive influence. If requested, the patient could receive midazolam in 1–2 mg for anxiety. If requested, the patient could receive fentanyl in 25 mcg increments for pain. Finally, if the patient’s pain and/or anxiety was uncontrolled even with fentanyl or midazolam, the patient could request IV sedation. A weight-based propofol bolus followed by propofol infusion was given for the remainder of the case. In addition to standard care, the VR group used a VR device (Occulus Go headset-Facebook Technologies, LLC) for immersive distraction. This group was instructed on how to use the VR device in the preoperative area, where they were given 20 minutes to familiarize themselves with the technology. A VR headset and a hand controller were given to allow patients to interact with the software (AppliedVR Inc). Patients could play a VR program of their choice in the preoperative area as well as during surgery. VR programs ranged from soothing landscapes (beach, historical cities, Grand Canyon, etc) accompanied by immersive sounds that simulated their environment. Patients could also choose to listen to an audio guide, in which a narrator could describe the significance and backstory of the chosen location. A member of the research team was available before surgery to teach the patient how to use the device and intraoperatively to help troubleshoot any issues with the headset. Figure 2 depicts a patient intraoperatively with the VR headset.

Pain was measured using the Numerical Rating Scale (NRS) ranging between 0 and 10, with 0 indicating no pain and 10 the worst pain imaginable. Anxiety was assessed similarly using the Numerical Visual Analog Anxiety Scale (NVAAS) ranging between 0 and 10, with 0 indicating no anxiety and 10 the worst anxiety imaginable. The NVAAS is an established anxiety scale and correlates in large part with the state-trait anxiety.^20^ This scale was chosen as it is easy to administer along with the NRS score for pain, given the fact that both scales had the same range. Measurements were made in the preoperative area after administration of local anesthetic, during surgery, and in the postanesthesia care unit (PACU). Intraoperative NRS and NVAAS scores were recorded at the beginning of the case and with the administration of all additional medications. The amount of midazolam, fentanyl, and propofol administered was recorded for the entire perioperative period. PACU recovery time, defined as the time to meet institutional discharge criteria, was also recorded. The VR group received an additional survey to assess their VR-device experience.

Prior study has shown a minimal clinically important difference (MCID) of 1.6–1.9 as well as a substantial clinical benefit of 2.2–2.6 for postoperative pain scores in nonshoulder hand and upper extremity.^21^ Thus, an effect size of 20% or a 2-point NRS difference between groups, a statistical power of 80%, and an alpha of 5% were chosen, and our target sample size was 34, with 17 control patients and 17 VR patients. No MCID related to NVAAS was available to support power calculations.

Comparisons between the control and VR groups were made using unpaired *t* tests for anxiety scores, pain scores, and medication use. Multivariable linear regression analysis was used to determine predictors of requiring additional analgesics or anxiolytics, controlling for covariates such as age (y), comorbidities, and other baseline demographic data. A *P* value of <.05 was considered statistically significant.

## Results

Forty-one patients completed the study protocol, with 21 patients in the control group and 20 patients in the VR group. As shown in Table 1, there was no difference in baseline characteristics, medical comorbidities, and preoperative vital signs between the two groups, except for age; the VR group was significantly younger than the control group (mean age, 46.3 vs 57.2 years; *P* = .01). Three patients withdrew from the study in the preoperative area prior to their VR experience.

**Table 1:** Baseline Characteristics

Variable	Non-VR (n = 21)	VR (n = 20)	*P* Value
Age (y)	57.5 (23–73)	46.3 (24–75)	.01∗
Sex (male/female)	10/11	7/13	.62
ASA Physical Status	1.8 (1–2)	1.6 (1–2)	.51
Height (cm)	168.4 (154.9–188)	167.8 (152.4–193)	.84
Weight (kg)	79.7 (53.5–120.1)	80.5 (50–129.3)	.90
Body mass index (kg/m^2^)	28 (19.2–37.3)	28.5 (20.8–39.8)	.78
History of high blood pressure	5	5	>.99
History of chronic nausea	1	0	>.99
Preoperative opioid use	1	2	.97
History of chronic pain syndrome	1	1	>.99
History of alcohol use disorder			
Active treatment for depression	2	6	.21
Active treatment for anxiety	2	7	.11
Procedures			
Carpal tunnel release	8	6	
Mass/cyst excision	4	3	
Trigger finger release	4	4	
Tendon repair	2	0	
Finger arthroplasty/arthrodesis	2	1	
Digit widget	1	1	
Fixation of finger fracture	0	2	
Removal of hardware	0	2	
Collateral ligament reconstruction	0	1	
3 Procedure duration	36.4 (9–104)	31.3 (13–76)	.50
Tourniquet used	8	4	.42
Systolic blood pressure (mm Hg)	135.3 (106–172)	132.1 (105–199)	.65
Diastolic blood pressure (mm Hg)	76.7 (63–98)	74.6 (49–95)	.55
Heart rate (bpm)	73.7 (50–102)	74.8 (63–98)	.78
Oxygen saturation (%)	97.5 (92–100)	98.6 (92–100)	.14

Values are reported as the mean (minimum–maximum) or the number of subjects (n), as indicated.

∗ *P* value < .05.

There were no significant differences in the pain levels between the VR and control groups during the preoperative, intraoperative, or postoperative periods (Table 2). Preoperative pain scores were low in both groups (VR 2.5 vs control 1.9; *P* = .45) and remained low before surgery (VR 1.3 vs control 1.1; *P* = .74) as well as after surgery in the PACU (VR 0.6 vs control 0.8; *P* = .64). Similarly, anxiety levels did not differ significantly between the groups at any time point. Preoperative NVAAS anxiety scores were comparable (VR 3.1 vs control 2.8; *P* = .73). During surgery, the VR group reported higher anxiety scores (VR 3.1 vs control 1.7), but this difference was not statistically significant (*P* = .09). Postoperative anxiety levels in the PACU were low for both groups (VR 0.3 vs control 0.7; *P* = .24). The PACU recovery time was similar between the groups, with no significant difference observed (VR 39.4 minutes vs control 46.3 minutes; *P* = .39).

**Table 2:** Pain and Anxiety Levels

Variable	Non-VR (n = 21)	VR (n = 20)	*P* Value
NRS pain level (before surgery)	1.90 (0–8)	2.5 (0–7)	.45
NRS pain level (during surgery)	1.1 (0–7)	1.3 (0–5)	.74
NRS pain level (PACU)	0 8 (0–6)	0.6 (0–5)	.64
Anxiety level (before surgery)	2.8 (0–8)	3.1 (0–8)	.73
Anxiety level (surgery)	1.7 (0–6)	3.1 (0–10)	.09
Anxiety level (PACU)	0.7 (0–5)	0.3 (0–2)	.24
Fentanyl (mcg)	27.4 (0–200)	10 (0–100)	.02∗
Midazolam (mg)	1.2 (0–4)	0.4 (0–4)	.04∗
Propofol (mg)	44.5 (0–323.9)	26.3 (0–401.2)	.54
PACU recovery time (min)	46.3 (15–140)	39.4 (17–70)	.39

Values are reported as the mean (minimum–maximum).

NRS, numerical rating scale.

∗ *P* value < .05.

The average amount of intraoperative propofol administered did not differ between the groups (VR 26.3 mg vs control 44.5 mg; *P* = .54). The VR group, however, received significantly less intraoperative fentanyl (VR 10 mcg vs control 27.4 mcg; *P* = .02) and midazolam (mean dose: VR 0.4 mg vs control 1.2 mg; *P* = .04).

In bivariate analyses, VR use was significantly associated with no midazolam administration (*P* = .01). In a multivariable model controlling for age, sex, preoperative opioid use, active treatment for anxiety or depression, and tourniquet use, VR use remained a significant predictor for no midazolam use (*P* = .02). In bivariate analyses, preoperative opioid use (*P* = .01) was the only variable that predicted fentanyl use. In a multivariable model controlling for the same variables listed above, preoperative opioid use remained a significant predictor for fentanyl use (*P* = .01). VR use was not a significant predictor of no fentanyl use in the multivariable model (*P* = .11).

Secondary outcomes focused on physiological responses during the perioperative period (Table 3). There were no significant differences between the groups in systolic and diastolic blood pressure changes at the time of incision, 30 minutes after incision, or in the PACU. Heart rate changes at the corresponding time points also did not differ significantly between the VR and control groups (all *P* > .05).

**Table 3:** Secondary Outcomes

Variable	Non-VR (n = 21)	VR (n = 20)	*P* Value
Change in systolic blood pressure (mm Hg)			
Incision	−5 (−20 to 17)	1 (−8 to 20.5)	.51
30min post incision	−7.5 (−20 to 2)	−5 (−21 to 1)	.87
PACU	1 (−13 to 24)	0.5 (−7.1 to 20.3)	.54
Change in diastolic blood pressure (mm Hg)			
Incision	−1.5 (−8 to 9)	−2.5 (−12.5 to 5)	.61
30min post incision	−3 (−14 to 4)	−2 (−13 to 3)	.44
PACU	2 (−8 to 20)	0 (−5 to 19)	.11
Change in heart rate (beats per minute)			
Incision	−1 (−11 to 15)	1.5 (−6 to 21)	.48
30min post incision	−2 (−7 to 6)	−0.5 (−11 to 5)	.90
PACU	0 (−8 to 3)	−1.5 (−7 to 6)	.89

Values are reported as the median (10th–90th percentiles).

**Figure 3:**
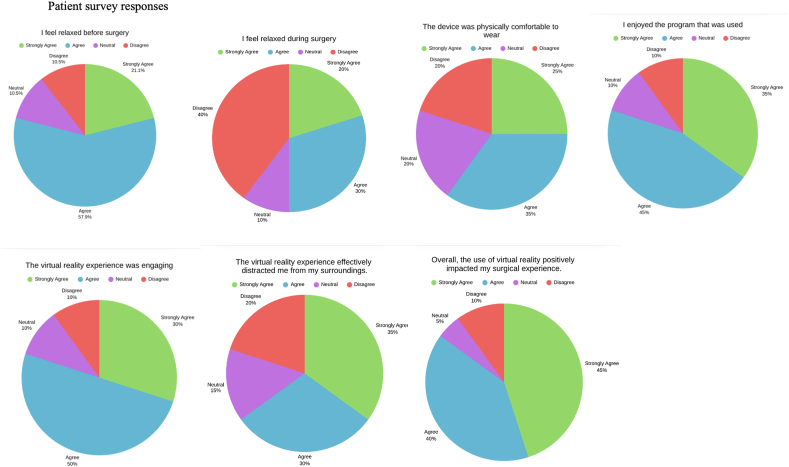
Post-treatment survey results of the VR treatment group.

Patient-reported outcomes from the VR group are summarized in Figure 3. Seventy-five percent (15/20) of patients reported feeling relaxed during surgery, and 60% (12/20) of patients found the device to be comfortable. Eighty percent (16/20) of patients enjoyed the VR program, and 80% (16/20) of patients found the program to be engaging and effective. As a result of the VR experience, 78% (14/20) of patients believed that their anxiety decreased, and 61.1% (11/20) of patients believed that their pain decreased. Overall, 85% (17/20) of patients believed that that the use of VR positively impacted their surgical experience. Although no patients reported any adverse events with VR usage, 4 (20%) of patients noted that the device was heavy.

## Discussion

This study illustrated the potential of VR as an adjunct to standard anesthetic care in patients undergoing minor hand surgery. Our findings demonstrated that although VR did not significantly alter patient-reported pain or anxiety levels or patient hemodynamics compared with the control group at any measured time point, it was associated with a significant reduction in the administration of intraoperative fentanyl and midazolam (*P =* .02, .04, respectively). Moreover, subjectively, 78% of patients felt like their anxiety decreased, and 61% of patients felt like their pain was decreased with the use of VR.

The application of VR as a non-pharmacological intervention to improve clinical outcomes has been supported by various studies in different medical settings.^22^, ^23^, ^24^, ^25^ To our knowledge, there are limited RCTs investigating VR implementation in the perioperative setting. An RCT conducted in 2022 by Chiu et al^26^ evaluated VR use in an ASC in Hong Kong, including cases within general surgery, endoscopic sinus surgery, transurethral resection, and arthroscopic surgery. They found that VR use in the preoperative area considerably decreased preoperative anxiety but found no differences in postoperative pain or length of stay. Notably, they used the Amsterdam Preoperative Anxiety and Information Scale, ranging from 6 to 30, which may have been more sensitive in detecting differences in anxiety level. Moreover, they did not report differences in the administration of anxiolytics or analgesics. In conjunction with the increased anxiolytic usage in non-VR patients in our study, it is conceivable that anxiety within the non-VR group was effectively treated by the midazolam, deflating what may have been higher NVAAS scores. This may also account for the relatively lower intraoperative NVAAS score in the control group. Notably, however, there was no difference in physiologic vital signs, indicating that although VR may not have completely matched the effect of midazolam, it was able to adequately control pain or anxiety to an acceptable degree. Thus, it may be reasonable to consider VR usage as a low-risk alternative to pharmacological antianxiolytic administration.

Within hand surgery, the ability to minimize analgesic use and risk has already been popularized with the increased use of wide-awake local anesthesia no tourniquet (WALANT) surgery within the past decade.^27^^,^^28^ Although WALANT eliminates the need for intravenous sedation, it is not an option for patients who cannot tolerate being awake during surgery. Patients with anxiety often cannot undergo WALANT as they need intravenous access for potential anxiolytic administration.^29^ There have been smaller case series and prospective studies evaluating VR use and its ability to reduce anxiety during WALANT.^18^^,^^30^, ^31^, ^32^, ^33^ We have previously showed in a case series of two patients who underwent WALANT that VR usage decreased perioperative anxiety and enhanced patient satisfaction.^32^ McCullough et al^31^ similarly evaluated 22 veterans undergoing WALANT and found lower anxiety scores with VR use. Hoxhallari et al^30^ compared VR with non-VR patients undergoing WALANT and noted decreased anxiety scores but no difference in pain or vital signs between groups. Similarly, they noted that 80% of their patients enjoyed the VR experience and would recommend it to others. Our study builds upon these prior works by not only evaluating patient-reported pain, anxiety, and satisfaction but also linking these findings to decreased need for administered anxiolytics. In our study, VR shows promise as an alternative “digital dose” of antianxiolytic, which may further qualify patients for the benefits of WALANT. Overall, the combination of WALANT and VR may further improve patient experience and perioperative outcomes.

Despite adequate regional anesthesia, ambulatory centers also offer intraoperative MAC, which patients may prefer to provide some pharmacological sedation. Patients undergoing MAC are still responsive to noxious stimuli and, thus, may require additional sedatives that may be augmented by VR use, as illustrated in our study. A similar trial conducted by Faruki et al^34^ compared patients undergoing hand surgery under MAC with patients under MAC as well as intraoperative VR. They found that the VR usage group received significantly less propofol compared with the control group. They found no differences in PACU pain scores, perioperative opioid usage, or postoperative functional outcome but did note significantly decreased PACU length of stay. Similarly, Alatarre et al^33^ evaluated VR use in patients undergoing upper-limb surgery, both emergent and elective, under peripheral nerve block and midazolam and showed decreased intraoperative anxiety score, increased patient satisfaction, and decreased hemodynamic changes. VR immersion can thus be considered a useful adjunct to a variety of different anesthesia options and a variety of upper-extremity surgery settings.

Although our study provides valuable insight into the use of VR in the perioperative setting, several limitations should be acknowledged. First, patients in the VR group were allowed to choose from a variety of immersive environments, and the specific content selected may have influenced their levels of relaxation and anxiety. Standardizing the VR content or conducting a more detailed analysis of how different VR environments impact patient experience could help address this limitation in future research. Second, the study focused on minor hand surgery, which typically requires lower amounts of sedative medications. Moreover, given the relatively small number of patients and the large range of minor hand surgeries, we were unable to control for different surgeries between the two groups. As such, our findings may not be generalizable to more complex or invasive surgical procedures in which there is a need for higher levels of sedation or longer operative times. Feasibility studies have been performed in orthopedic procedures such as joint replacement, but larger randomized controlled trials are necessary.^35^, ^36^, ^37^ Furthermore, although our sample size was based on power calculations for MCID of pain, we may have been underpowered to detect differences in other factors such as NVAAS for hemodynamic changes. Third, our study was not blinded in that the patient, the anesthesiologist, and the surgical team were aware of the assigned treatment. It is certainly possible that the anesthesiologist may have been biased to administer lower doses of medication as a result of observing the VR device on the patient. Nonetheless, we do not believe that this diminishes the potential benefits of VR. The patients reported high satisfaction and low-anxiety and low-pain scores despite lower medication dosages in the VR group, which is the overall goal of perioperative care. Fourth, we were limited by institutional policies at our ASC. Specifically, most patients who were ASA class III did not qualify for surgery at the ASC, and because of the established workflow at our high-volume ASC, we were limited in our ability to involve anesthesia or administer sedation in the preoperative area. At our institution, the local anesthetic is typically administered by the surgical team in the preoperative holding area at least 30 minutes prior to surgery in order to allow for adequate anesthesia and epinephrine effect. The preoperative area is not set up for routine anxiolytic administration, and therefore, we would not have been able to collect medication data during local injections. Thus, we elected to limit the use of VR technology to the surgery alone to standardize the procedures. We recognize that the use of VR during the administration of local anesthesia could certainly amplify its beneficial effect and should be considered in future studies. Finally, the significant age difference between the VR and control groups may have influenced the results, as younger patients might interact differently with VR technology compared with older patients. Although we controlled for age in our analyses, future studies should aim for a more balanced age distribution to minimize potential confounding effects.

Our study contributes to the growing evidence supporting the use of VR as a nonpharmacological adjunct in perioperative care. The considerable reduction in intraoperative midazolam administration in the VR group suggests that VR can enhance patient comfort and reduce the need for anxiolytic medications during minor hand surgery. Although we did not observe significant differences in pain and anxiety levels, the overall positive feedback from patients regarding their VR experience indicates potential benefits in patient satisfaction. This study serves as a proof of concept of how VR could play a important role in enhancing personalized care approaches in the perioperative setting.

## Conflicts of Interest

No benefits in any form have been received or will be received related directly to this article. The authors have no relevant conflicts to disclose.
