# Supplementary material for: Digital Doses: Virtual Reality Use for Perioperative Pain and Anxiety in Patients Undergoing Hand Surgery
Source: J Hand Surg Glob Online. 2025 Sep 23;7(6):100830. doi: 10.1016/j.jhsg.2025.100830 (PMC12492192; doi:10.1016/j.jhsg.2025.100830)
Supplement: Supplementary Material [file mmc1.docx]

**Figure S1**. Handout given to the anesthesia providers at the ambulatory surgery center

**17-22021 Outline**

**Aims:**

1. Compare anxiety and pain levels between VR + Control groups
2. Compare amount of medications given for anxiety and pain between VR + Control groups

**Study Subjects**: Patients having a small hand/arm surgery under local/MAC. NO General Anesthesia and NO regional anesthesia.

- Patients who have met inclusion criteria and who have agreed to participate in the study will be randomly assigned to the VR vs. control groups the day of the procedure.
- A member of the research team will orient the patient to the VR device if they fall into the VR group.
- A member of the research team will be with the patient in pre-op and intraop for both research groups. The member of the research team can help with bringing the patient into the room, place monitors and to position the patient, but will not administer anesthesia.
- Both the VR group and control group will get local anesthesia and MAC as outlined below. Since anesthesia providers have different threshold and standards as to when they administer sedation, it is VERY important to follow the anesthesia administration standards below, within reason, to be able to accurately assess the effect of the VR intervention.

**VR Group**: VR, local anesthesia and no IV sedation unless requested by patient.

Patients who have been assigned to this study group will be oriented to the VR goggles and headsets in Pre-op. Use of VR will commence in pre-op and continue intraoperatively. A member of the team will be present in preop and intraop to place the VR device on the patient and troubleshoot any problems.

**CONTROL GROUP**: No VR, local anesthesia and no sedation unless requested by patient. Patients who have been assigned to this group will get the same anesthesia care as the VR group without the VR intervention. Sedation is not automatically given unless requested by the patient.

**Guidelines for the Administration of Anesthesia**

- When consenting the patient, the type of anesthesia should be described as local (if applicable) with MAC. State that IV sedation will only be administered if requested by the patient. The patient should expect to get no sedation unless “she really needs it.”
- Patient they can request sedation at any point. Anesthesia provider routinely asks the patient how they are doing (every 15 minutes), but do not offer medications directly to avoid this suggestion from influencing their request for sedatives. If the patient is overly anxious or uncomfortable, they can be more directly asked if they would like specific IV medications.

e

**Medication administration guidelines:**

Pre-op: Midazolam 1-2mg PRN only if requested.

Intra-op:

- If requested: Midazolam 1-2mg for anxiety only. Fentanyl 25mcg increment for pain only, titrate as needed.
- If Midazolam +/- Fentanyl are not enough, administer Propofol bolus +/- Propofol Infusion. Uptitrate slowly.
- Measure blood pressure at least every 3 minutes.
- Zofran prn.

Post-op: If requested. Fentanyl PRN in 25 mcg increments.
